# Risk of death after type of intimate partner violence (IPV) involvement

**DOI:** 10.23889/ijpds.v9i5.2795

**Published:** 2024-09-18

**Authors:** Ann Raymond, Toby Watt, Laurie Rachet-Jacquet, Hannah-Rose Douglas, Anna Head, Chris Kypridemos

**Affiliations:** 1 REAL Centre, Health Foundation; 2 Department of Public Health, Policy and Systems, University of Liverpool

## Objective and Approach

Most studies have focused on women’s IPV victimization but perpetration and bi-directional violence remain understudied. Using linked criminal justice and population registers in Manitoba, Canada, we assessed the risk of death according to the role in IPV incidents (accused-only, victim-only, bidirectional, none). In this retrospective matched cohort study, we assembled a cohort of 212,068 adults who were followed from April 2004 to March 2023 to assess IPV incidents and subsequent death. Those involved in an IPV incident were 1:3 matched to persons with no history of IPV based on birth year, sex and marital status at the time of the incident.

## Results

Men comprised 85% of accused-only, 21% of victim-only, and 50-51% in the other two groups. Overall, compared to those without IPV involvement, the adjusted Hazard Ratios for all-cause mortality were 1.38 [95% Confidence Intervals (CI): 1.28, 1.49] for accused-only, 1.39 (1.29, 1.50) for victim-only and 1.45 (1.19, 1.77) for bidirectional IPV. The associations were stronger among women, particularly that of bidirectional violence [1.24 (0.97, 1.60) among men and 1.92 (1.39, 2.66) among women]. Similar patterns were found for intentional mortality [0.96 (0.56, 1.64) among men and 2.43 (1.27, 4.65) among women].

## Conclusions

There are clear sex inequities in IPV involvement. Any type IPV involvement is associated with higher risk of death, particularly among women, who comprise most of the victims.

## Implications

Use of linked criminal justice data allows studying the consequences of IPV victimization, perpetration and bidirectional violence and monitor gender inequities over time.

